# Identifying Water-Salt Homeostasis and Inflammatory Response in Pathological Cardiac Surgery-Associated Acute Kidney Injury: NT-proBNP-related lncRNAs and miRNAs as Novel Diagnostic Biomarkers and Therapeutic Targets

**DOI:** 10.7150/ijms.107589

**Published:** 2025-01-21

**Authors:** Bianfang Li, Jianhui Wang, Yuchen Gao, Xiaojing Wu, Chunrong Wang, Jin Wang, Jia Liu, Yuefu Wang

**Affiliations:** 1 Department of Surgery Intensive Care Unit, Emergency and Critical Care Medical Center, Beijing Shijitan Hospital, Capital Medical University, Beijing, 100038, China.; 2 Department of Anesthesiology, Fuwai Hospital, National Centre for Cardiovascular Diseases, Chinese Academy of Medical Sciences and Peking Union Medical College, Beijing, 100037, China.; 3 Xianning Medical College, Hubei University of Science & Technology, Xianning 437000, China.

**Keywords:** CS-AKI, NT-proBNP, lncRNA, miRNA, RNA-sequencing

## Abstract

Acute kidney injury related to cardiac surgery (CS-AKI) is a serious medical issue that creates significant social and economic challenges globally. Inflammatory responses and disruptions in water and salt balance are important contributors to CS-AKI. Earlier studies indicated that pre-surgery levels of NT-proBNP were a dependable indicator of CS-AKI. Emerging evidence indicates that the abnormal expression of microRNA (miRNA) and long non-coding RNA (lncRNA) plays a role in the occurrence of CS-AKI. However, the important roles and mechanisms by which NT-proBNP affects lncRNA and miRNA in CS-AKI are still unclear. Here, we investigated lncRNA and miRNA expression patterns in BNP-high, BNP-stable, AKI, and non-AKI groups through whole transcriptome sequencing analysis. The BNP group exhibited differential expressions of 105 miRNAs and 138 lncRNAs. We identified 7 common miRNAs and lncRNAs in both the BNP and AKI groups. A functional and pathway enrichment analysis of the target genes associated with these miRNAs and lncRNAs was conducted, indicating that miR-135a-5p, miR-138-5p, miR-143-3p, and miR-206 are key factors in CS-AKI, particularly in regulating inflammatory responses and water-salt balance. These results provide fresh perspectives on research directions and possible treatment approaches for CS-AKI.

## 1. Introduction

Acute kidney injury following cardiac surgery (CS-AKI) is a prevalent and serious issue, affecting about 40% of patients and resulting in significant mortality^1^, ^2^. Recent research has pointed out that disruptions in water and salt balance, along with inflammation, play a role in AKI^3^-^5^. Numerous clinical studies have identified a link between pre-surgery NT-proBNP and BNP levels and the occurrence of CS-AKI, particularly in severe cases, with NT-proBNP significantly improving CS-AKI prediction^6^-^8^. Currently, our comprehension of the relevant molecular mechanisms between CS-AKI and NT-proBNP is rather restricted.

Non-coding RNAs (ncRNAs), prevalent in the human genome, are crucial for gene regulation^9^ and hold potential as biomarkers for diagnosing diseases like AKI^10^. Research indicates that the improper regulation of ncRNAs is linked to the pathological development of CS-AKI^11^-^14^. Within the group of ncRNAs, lncRNAs and miRNAs are crucial components that need to be studied in relation to CS-AKI^9^, ^10^. Exploring the link between NT-proBNP and CS-AKI through lncRNAs and miRNAs is crucial, as it could lead to more effective treatments for CS-AKI.

Our past findings showed that the NT-proBNP prior to operation, when elevated, had a connection with a boosted possibility of CS-AKI. Therefore, the current research concentrated on exploring the ncRNA alterations in expression among participants diagnosed with CS-AKI and probing into potential regulation modes via RNA sequencing.

## 2. Materials and Methods

### 2.1 Patients and ethics approval

This cohort study included 30 participants who underwent heart surgery at Fuwai Hospital in Beijing, China. Individuals with mental disorders, significant liver and kidney issues, a history of major surgeries excluding cardiac surgery, or those who declined to join the trial were not included. The study procedure was supervised and approved by Fuwai Hospital's Institutional Review Board (IRB), which waived the requirement for written informed consent due to the retrospective nature of the research. The study was carried out in full compliance with the applicable regulations and guidelines.

### 2.2 Gathering samples and data origins

Healthcare professionals documented the patients' fundamental and clinical information, encompassing demographic details, biochemical markers, and data from before, during, and after surgery. Samples of serum and urine were gathered at different times during the after-surgery phase (0, 12, 16, 24 hours) and before surgery^15^, ^16^. As mentioned in our earlier article^6^, the hospital laboratory regularly measured serum NT-proBNP levels before and after surgery. Additionally, we periodically monitor plasma levels of biochemical markers.

### 2.3 Group division

Patients were categorized into two groups according to NT-proBNP values: patients whose post-surgical to pre-surgical specific value was equal to or greater than 2 were categorized into the BNP-high group. On the other hand, patients whose specific value was less than 2 were assigned to the BNP-stable group. Besides, among the 30 participants, those who were diagnosed with AKI were categorized as AKI, while the rest were classified as non-AKI. The patients had their CS-AKI diagnosed as per the diagnostic criteria defined in the Kidney Disease Improving Global Outcomes (KDIGO) guidelines^17^.

### 2.4 RNAseq

TRIzol (Thermo Fisher Scientific, USA) method was applied to isolate RNA. The NanoDrop ND-1000 (Nano Drop, Wilmington, DE, USA) was used to quantify the RNA concentration and quality of each sample. The RNA integrity was analyzed by the Agilent 2100 Bioanalyzer (Agilent Technologies, USA). The Collibri Stranded RNA Library Prep Kit (Thermo Fisher Scientific, USA) was used for mRNA library preparation. Afterward, PCR was employed to enrich DNA fragments followed by library purification and validation. RNA sequencing was performed on the Illumina NOVA 6000 platform^18^-^20^.

The NEBNext® Multiplex Small RNA Library Prep Set for Illumina (NEB, USA) was used to establish the small RNA library. In this procedure, 3' adapters tailored for microRNAs and other small RNAs were ligated to RNA molecule ends, followed by the addition of 5' adapters. Single-strand cDNAs were amplified using RT-PCR and subsequently purified through gel electrophoresis. The quality of the cDNA construct was confirmed using the Agilent 2100 Bioanalyzer. Using the cBot (Illumina, USA), cluster generation was completed. Finally, the small RNA library underwent sequencing on the same sequencing platform as the mRNA library^21^-^24^.

### 2.5 Bioinformatics analysis

High-quality data were acquired by filtering the raw next-generation sequencing reads using Seqtk (https://github.com/lh3/seqtk). Under the guidance of the Ensembl GTF gene annotation file, the Cuffdiff software was utilized to obtain the FPKM (Fragments per kilobase of exon per million fragments mapped) values of mRNA at the gene level and small RNAs (including miRNAs and lncRNAs). These FPKM values served as the expression profiles of mRNA and small RNAs. Subsequently, the fold change and P-value between the two groups of samples were calculated to screen for differentially expressed mRNAs, miRNAs, and lncRNAs. For mRNAs, GO (Gene Ontology) and KEGG (Kyoto Encyclopedia of Genes and Genomes) pathway analyses were conducted directly. Meanwhile, the target genes of miRNAs and lncRNAs were predicted, and then GO and KEGG pathway analyses were performed on these target genes as well. Distinct lncRNAs and miRNAs were identified in both the AKI and BNP groups, and their overlaps were illustrated using Venn diagrams^25^-^29^.

### 2.6 Statistics

Variables following a normal distribution were analyzed using a Student's t-test, with results presented as mean ± standard deviation (SD). Normality tests were performed on continuous variables for data analysis. The Mann-Whitney U test was used to analyze non-parametric data not following a normal distribution, with results presented as medians and interquartile ranges (IQRs). Categorical variables were analyzed using either Fisher's exact test or the χ² test, with results presented as numbers (%). For the continuous variables, Pearson correlation analysis was applied when the data exhibited a normal distribution; otherwise, Spearman correlation analysis was utilized^30^-^33^. Statistical significance was set at a P-value ≤ 0.05.

## 3. Results

### 3.1 Population characteristics

**Figure 1:**
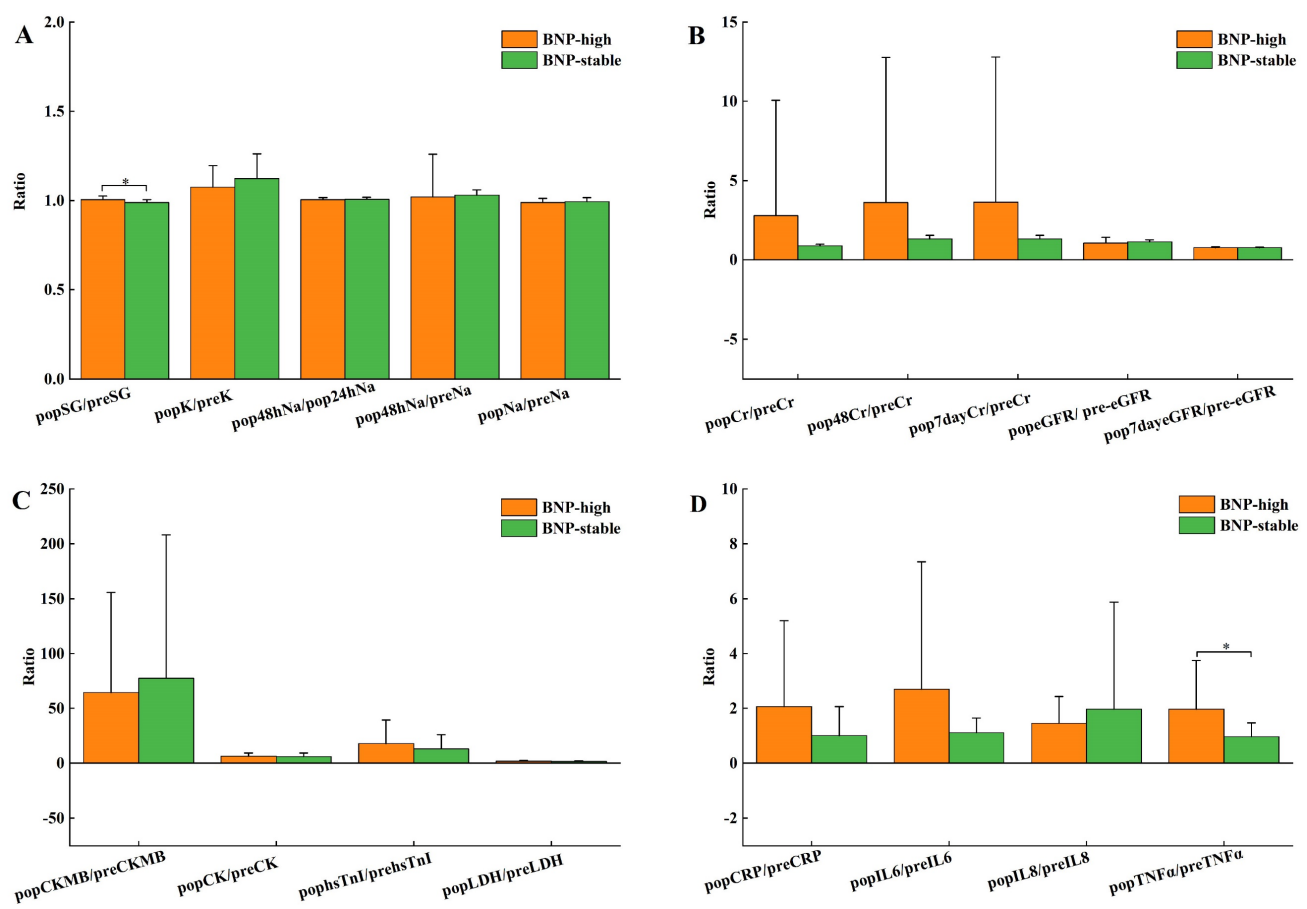
Comparison of after-surgery and pre-surgery ratios of related biochemical indexes in BNP high group and BNP stable group, pop, after-surgery; pre, pre-surgery. (A) Metabolism of body fluids and salts K, Na, SG, Urine specific gravity; (B) Renal function Cr, Creatinine; eGFR, estimated glomerular filtration rate; (C) Cardiac function CK-MB, creatine kinase isoenzymes; hsTnI, high sensitivity troponin I; LDH, lactic dehydrogenase; IGF1,insulin-like growth factor 1; (D) Inflammatory factors CRP, C-reactive protein; IL6, Interleukin-6; IL8, Interleukin-8; TNFα, tumor necrosis factor-α. *P<0.05 comparison between BNP-high group and BNP-stable group.

Table 1 shows no remarkable differences in baseline data, details within the operation, and AKI incidence after the operation between BNP groups. Pre-surgery NT-proBNP levels of the BNP-high group were dominantly decreased compared to the BNP-stable group (*P*<0.001), with biochemical indicators showing no noticeable distinctions, as shown in Table 2. As shown in Table S1, the NT-proBNP fold change was 9.69 (8.11-12.81) for the BNP-high group and 1.19 (0.91-1.47) for the BNP-stable group. Still, there were no significant discrepancies in other heart function markers (Figure 1C). Figure 1 compares the pre- and post-surgery biochemical index ratios in these 2 BNP groups. In contrast to the group with stable BNP levels, the group with high BNP levels demonstrated substantially elevated SG proportion before and after surgery (Figure 1A). Renal function did not show any statistically significant changes across the groups (Figure 1B). The comparison of inflammatory factors between groups revealed that the ratios of TNF and IL10 at 24 hours after surgery and pre-surgical were significantly higher in the BNP-high group compared to the BNP-stable group (Table S1, Figure 1D).

Spearman correlation analysis revealed no significant correlation between BNP multiple and water-salt metabolism and inflammation indexes (Table 3). TNFα T5/T1 demonstrated a prominent medium positive association with inflammatory-promoting elements IL-6, CRP, and IL-8, as well as with the anti-phlogistic element IL-10 (r=0.574, P=0.001).

### 3.2 DELs across various groups

**Figure 2:**
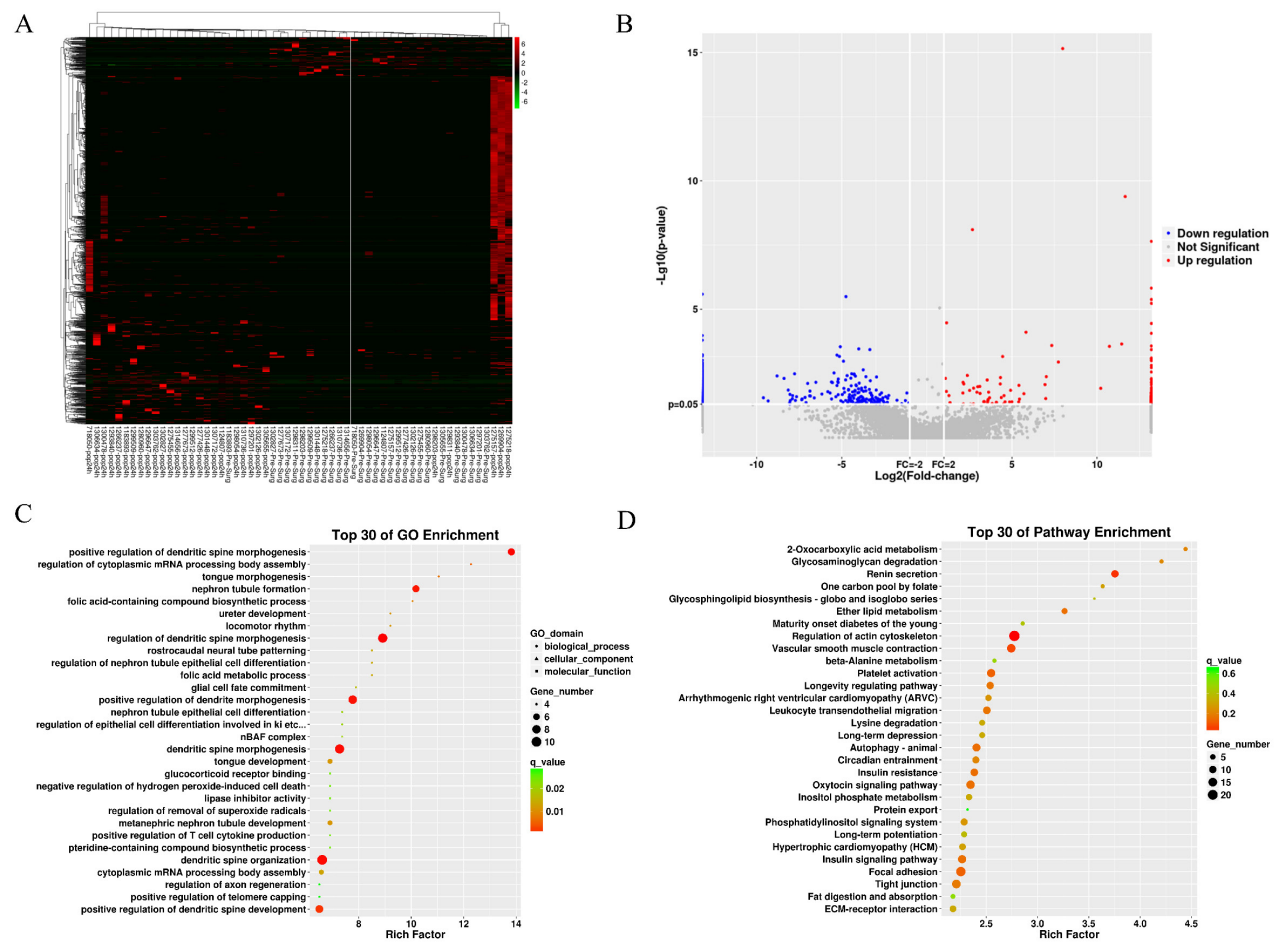
The differential lncRNA of BNP grouping. (A) heatmap of differential lncRNA. (B) volcano plot of differential lncRNA. (C) GO term enrichment plot of differential lncRNA target genes. (D) KEGG pathway enrichment plot of differential lncRNA target genes.

Table S2 shows QC results of lncRNA sequencing and miRNA sequencing. Figures 2A and 2B present lncRNAs with differential expressions (DELs) between 2 BNP groups. Among the 138 DELs found, 108 exhibited up-regulation and 30 exhibited down-regulation. The predicted differential lncRNA target genes underwent GO term and KEGG pathway enrichment analysis, with results displayed as scatter plots (Figures 2C and 2D). According to the top 30 enriched GO terms, the target genes were chiefly engaged in regulating dendritic spine morphogenesis (Figure 2C). The top 30 KEGG pathways showed significant enrichment in pathways associated with renin secretion and actin cytoskeleton regulation (Figure 2D). The supplementary materials (Figure S1) include the differential lncRNAs identified for the AKI group and the GO and KEGG analysis results of the target genes.

### 3.3 Differential expression of miRNAs across various groups

**Figure 3:**
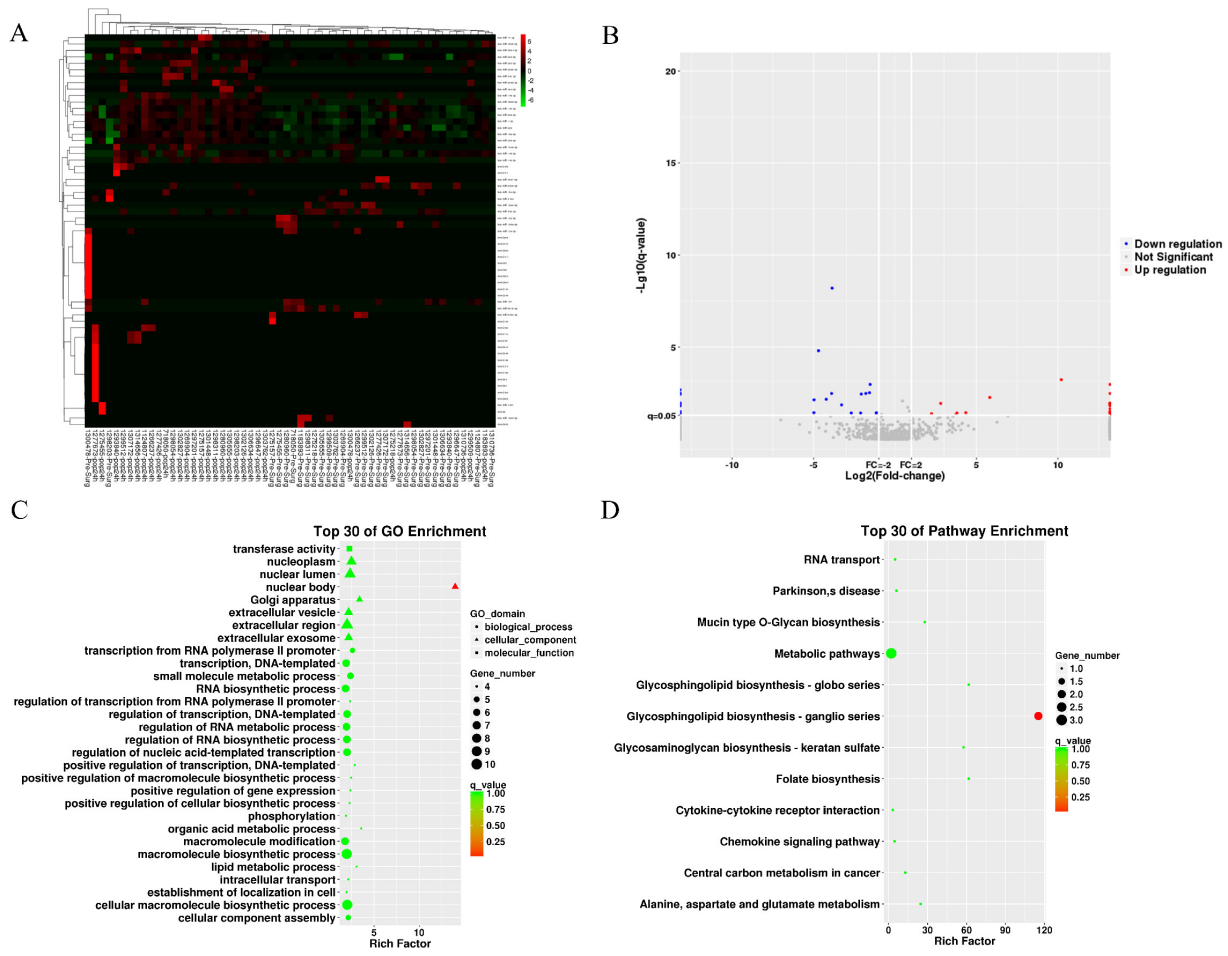
The differential miRNA of BNP grouping. (A) heatmap of differential miRNA. (B) volcano plot of differential miRNA. (C) GO term enrichment plot of differential miRNA target genes. (D) KEGG pathway enrichment plot of differential miRNA target genes.

We collected 20 MB of raw small RNA sequencing data per sample (QC results in Table S2). The heatmap and volcano plot show differentially expressed miRNAs (DEMs) identified by edgeR in the BNP groups (Figures 3A and 3B), revealing 62 up-regulated and 43 down-regulated miRNAs. Forecasted genes targeted by DEMs underwent GO and KEGG analysis, of which the results are displayed in Figures 3C and 3D. GO analysis indicates target genes are mainly involved in macromolecule and RNA biosynthesis/modification and are located in nuclear components. KEGG analysis shows significant clustering in metabolic pathways and glycosphingolipid biosynthesis-ganglion series. Similar analyses were conducted for the AKI group.

### 3.4 Common DEMs and DELs in different groups

**Figure 4:**
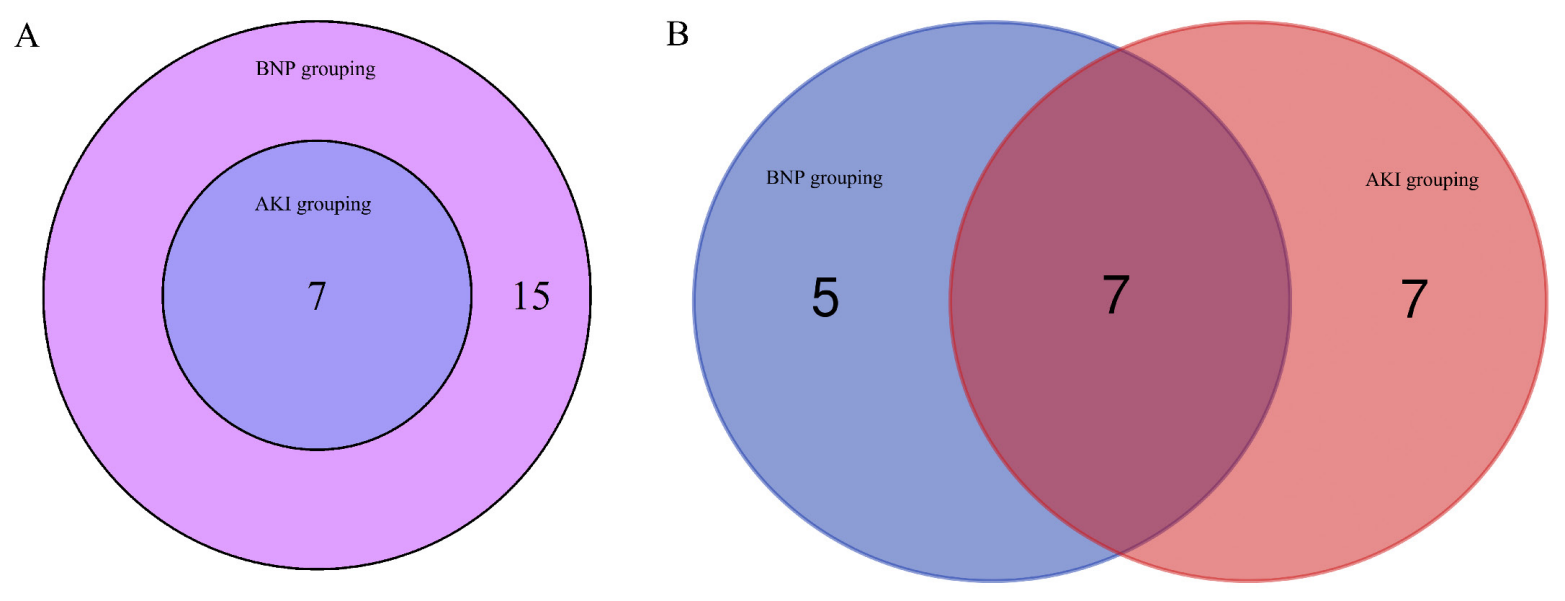
The common lncRNA and miRNA of BNP grouping and AKI grouping by venn diagrams. (A) The common lncRNA of BNP grouping and AKI grouping by venn diagrams. (B) The common miRNA of BNP grouping and AKI grouping by venn diagrams.

**Figure 5:**
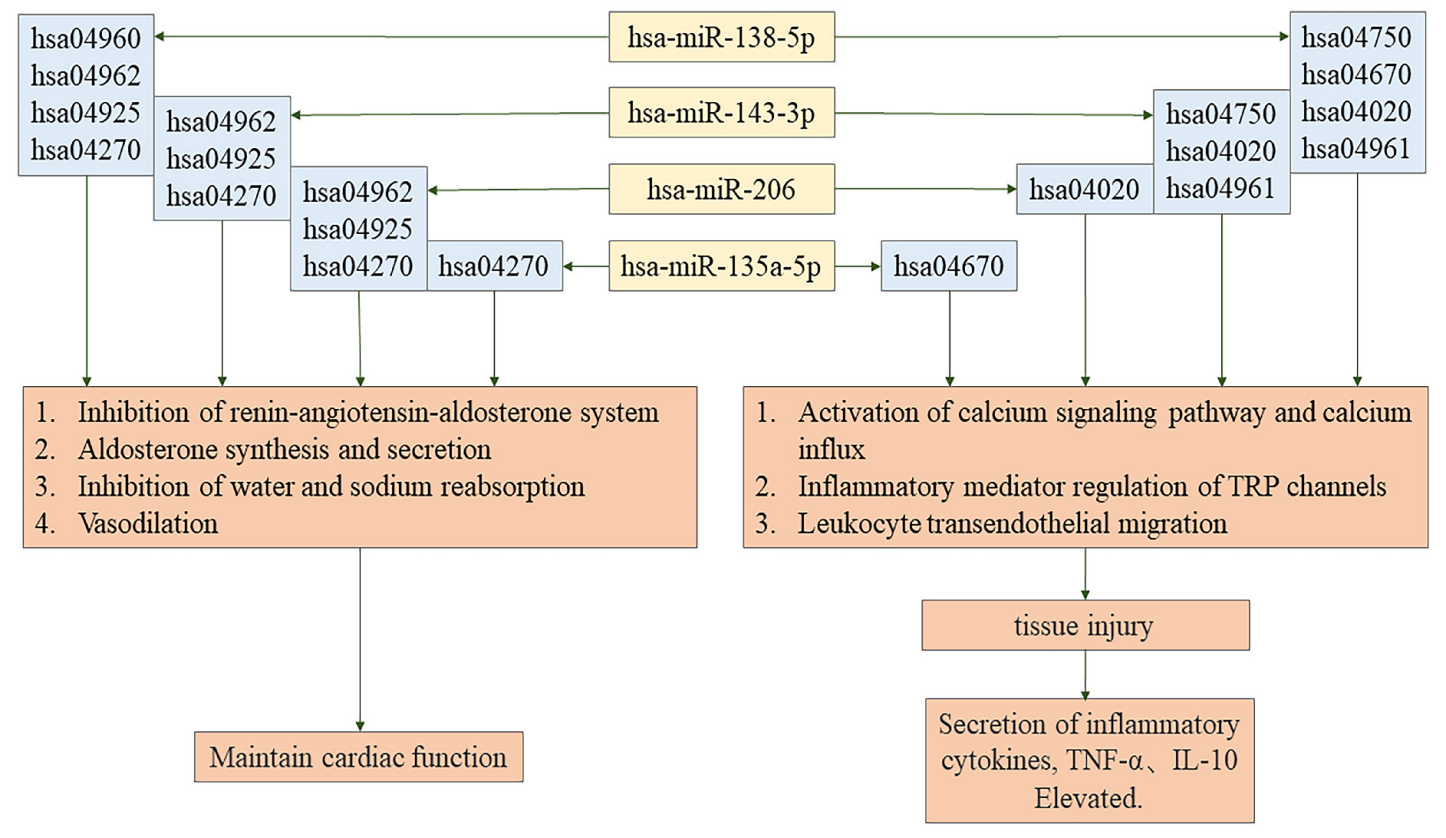
The regulatory pathway of hsa-miR-135a-5p, hsa-miR-138-5p, hsa-miR-143-3p, hsa-miR-206.

Seven miRNAs as well as seven lncRNAs were screened out by Venn diagrams. Among them were four novel lncRNAs, namely MSTRG.129696.18, MSTRG.39610.1, MSTRG.129293.3, and MSTRG.129696.10. These are graphically depicted in Figure 4 to afford additional elucidation and serve as a referential resource. Table 4 displays their regulation status. The 7 lncRNA target genes screened out showed no association with pathways associated with AKI or BNP rise. Among the 3 already identified lncRNAs that are transcribed from exons, high expression levels in the kidney and heart, however, are shown only by NON-HSAT160556.1. BNP and AKI pathways are closely involved with 4 miRNAs, namely hsa-miR-206, hsa-miR-138-5p, hsa-miR-135a-5p, and hsa-miR-143-3p (Figure 5).

## 4. Discussion

The pre-surgical serum levels of NT-proBNP are broadly acknowledged as predictors for CS-AKI^6^, ^8^. Nevertheless, the precise pathophysiological mechanisms and molecular regulatory pathways underlying CS-AKI remain inadequately elucidated. It is postulated that disruptions in the water and salt homeostasis related to BNP, in conjunction with inflammatory responses, are intimately correlated and play a crucial role in both the onset and the advancement of CS-AKI^3^-^5^.

In both BNP groups, our study made it clear that there were notable differences in urinary specific gravity quotient between the after-surgery and pre-surgical periods, thus highlighting variations in the metabolism of body fluids and salts. No significant differences were found in serum potassium and sodium levels. As BNP reduces sodium reabsorption in the renal inner medullary collecting duct without affecting water reabsorption^34^, we suggest that the group with high BNP levels showed higher urinary specific gravity due to reduced reuptake of sodium.

As a crucial effector organ, the kidney plays an essential role in keeping the homeostasis of the metabolism of body fluids and salts. Biotic elements influencing this internal balance can significantly impact renal function, with inflammatory mediators being particularly notable. Studies on marathon runners suggest that AKI is linked to sodium and water loss through sweat and increased serum copeptin levels^35^, ^36^. Marathon runners undergo sustained physical exertion, which induces an acute inflammatory response marked by elevated cytokine levels, including TNF-α, IL-6, and IL-8^35^, ^37^. Disruptions in cellular water and salt homeostasis can elevate tonicity beyond tolerable thresholds, thereby exacerbating inflammatory responses and inducing cellular apoptosis^38^. BNP plays a vital and coordinating role between cardiac and renal functions, particularly with regard to the modulation of inflammatory responses, sodium excretion, and the maintenance of water balance^34^, ^39^. It is widely acknowledged that inflammation has an intricate link with BNP and CS-AKI^34^, ^40^-^43^. A growing body of evidence suggests that elevated plasma concentrations of TNF-α, IL-6, IL-8, and IL-10 are significantly involved in the pathophysiology of CS-AKI. Consistent with the current literature, it is indicated by our findings that the ratios of IL-10 and TNF-α are significantly increased 24 hours following surgery in contrast to the pre-surgery in the group of high BNP levels. RNA sequencing of both 24-hour pre-surgical and post-surgical plasma samples was executed to investigate the potential pathways associated with CS-AKI. It is indicated by the results that in reply to NT-proBNP, the progression of CS-AKI might involve various inflammatory mediators, which demands more research for clarification of the interactions and underlying pathways.

Through GO and KEGG pathway enrichment analyses, we have obtained important findings in CS-AKI patients characterized by increased BNP levels. We noticed that there were rather significant differences in the expressions of 7 microRNAs and 7 lncRNAs before and after the operation. Specifically, 4 of these miRNAs have demonstrated crucial roles. On the one hand, they are involved in regulating the homeostasis of body fluids and salts. For example, miR-143-3p, miR-206, and miR-138-5p are predicted to be involved in the inhibition of water reabsorption (corresponding pathway: hsa04962). Also, they are predicted to participate in sodium reabsorption (corresponding pathway: hsa04960), aldosterone synthesis and secretion (corresponding pathway: hsa04925), and vasodilation (corresponding pathway: hsa04270). On the other hand, they regulate the secretion of inflammatory factors, and these inflammatory factors play a significant role in regulating tissue damage. In addition, miR-135a-5p is associated with vasodilation (hsa04270). Particularly, it is the reduction of sodium intake that can lead to the upregulation of the level of miR-143-3p among patients with untreated hypertension^44^. Moreover, it has been confirmed that miR-206 can regulate the homeostasis of Na⁺ by targeting NCX2^45^. What these results suggest is that these miRNAs might genuinely be involved in the modulation of water and sodium metabolism, which means deeper exploration is needed.

This research indicates that miR-143-3p and miR-138-5p may influence inflammatory responses by regulating mediators linked to TRP channels and activating calcium signaling pathways and calcium influx. These findings are consistent with earlier studies showing elevated plasma levels of miR-143-3p in AKI caused by drugs and the dual role of miR-138-5p in modulating inflammatory responses in different diseases^46^-^48^. Variability in miR-138-5p expression and function across pathological contexts may result from individual patient differences and sample selection criteria^49^-^51^.

Recent studies have identified miR-135a-5p as being downregulated in patients with atherosclerosis, where it has been implicated in promoting inflammatory responses and oxidative stress^52^-^55^. Meanwhile, miR-135a-5p has also been found to be decreased in smooth muscle cells of the human aorta, and in this case, it can alleviate vascular inflammation in rats with chronic kidney disease^56^-^62^. In this research, it was determined that miR-135a-5p exhibited a remarkable downregulation in both the AKI and BNP groupings and was linked to the process of migration of leukocytes across the endothelium (hsa04670). miR-206 contributes to inflammatory responses and increases the reactive oxygen species (ROS) of mice via targeting as well as inhibiting SOD1^63^-^67^. Overexpressed miR-206 increases inflammatory-stimulating immunomodulators IL-1β, IL-6, and CCL5^68^-^70^. In the present research, a connection was uncovered between miR-206 and calcium influx along with the activation process of the calcium signaling pathway (hsa04020)^71^. Additionally, miR-143-3p, miR-206, miR-135a-5p, and miR-138-5p likely modulate inflammatory cytokine secretion and tissue damage, supporting their documented roles in existing literature^72^-^77^. The observations demonstrate the four miRNAs screened out might influence the inflammatory reaction in the kidney and the metabolism of body fluids and salts, indicating their potential intermediary role between NT-proBNP and CS-AKI.

As an inflammation marker for the kidneys and myocardium, lactate dehydrogenase (LDH), mainly detected in the myocardium and kidneys^78^-^80^, shows increased serum activity due to cell lysis or membrane disruption^78^-^82^. Studies show that elevated serum LDH levels in patients with AKI or CS-AKI suggest its potential as a predictive biomarker for CS-AKI^81^, ^82^.

## 5. Conclusion

Our study found no significant differences in nephric function or AKI incidence changes amid pre-surgical and after-surgery groups with stable or increased BNP levels. The study indicates that existing cardiac circulatory arrest procedures are generally safe, and the surgery-induced rise in NT-proBNP levels does not worsen renal function impairment. In larger cohorts, patients with pre-surgical elevated NT-proBNP levels show a higher incidence of AKI after cardiac circulatory arrest surgery, seemingly independent of operation. Pre-surgical low cardiac function or pre-existing renal impairment might account for this.

MicroRNAs, including miR-138-5p, miR-143-3p, miR-135a-5p, and miR-206, impact the regulation of the inflammatory response to tissue injury and sodium and water metabolism. These microRNAs may impact NT-proBNP metabolism and AKI through inflammatory response factors, potentially regulating AKI in the BNP-high group. Future research shall clarify heart-kidney injury mechanisms and discover prospective treatment and prophylaxis loci.

Generally, this study highlights the impact of DELs on CS-AKI. There is an intimate correlation between DEMs and inflammatory response and water-salt stability, offering significant insights into the molecular mechanisms of CS-AKI. These findings establish a foundation for the exploration of novel molecular markers indicative of early renal dysfunction, thereby facilitating the development of innovative CS-AKI treatment.

## Supplementary Material

Supplementary figures and tables.

## Figures and Tables

**Table 1:** Comparison of baseline data grouped according to BNP multiples.

Variables	BNP-high(n=15)	BNP-stable(n=15)	*P*-value
Age, year	59.87±8.14	54.87±15.22	0.275
Male, n (%)	10(66.67%)	13(86.67%)	0.196
BMI, (kg/m^2^)	25.59±3.25	24.10±2.93	0.196
Smoking, n (%)	9(60%)	10(66.67%)	0.624
Diabetes mellitus, n (%)	3(20%)	2(13.33%)	0.624
CHD, n (%)	9(60%)	7(46.67%)	0.464
Hyperlipidemia, n (%)	6(40%)	7(46.67%)	0.713
Hypertension, n (%)	11(73.33%)	8(53.33%)	0.256
Heart failure, n (%)	0(0)	1(6.67%)	0.309
Asthma, n (%)	1(6.67%)	0(0)	0.309
PVD, n (%)	1(6.67%)	2(13.33%)	0.543
cerebral infarction, n (%)	0(0)	1(6.67%)	0.309
Previous surgery, n (%)	5(33.33%)	8(53.33%)	0.269
Previous cardiac surgery, n (%)	1(6.67%)	4(26.67%)	0.142
Pulmonary hypertension, n (%)	1(6.67%)	4(26.67%)	0.142
Liver insufficiency, n (%)	0(0)	1(6.67%)	0.309
LVEF (%)	61.80±5.57	60.27±7.51	0.530
RWMA, n (%)	5(33.33%)	3 (20%)	0.409
Surgery			
CPB, n (%)	10(66.67%)	10(66.67%)	1.000
CPB duration (min)	73(0-101)	68(0-150)	0.567
Blocking duration(min)	50(0-76)	43(0-95)	0.683
Pre-surgical anticoagulants, n (%)	8 (53.33)	4 (26.67%)	0.136
Pre-surgical statins, n (%)	8 (53.33%)	6 (40%)	0.464
After-surgery			
After-surgery atelectasis, n (%)	9 (60%)	7 (46.67%)	0.464
after-surgery pleural effusion, n (%)	10(66.67%)	12(80%)	0.409
Arrhythmia after surgery, n (%)	2(13.33%)	6(40%)	0.099
after-surgery atrial fibrillation, n (%)	2(13.33%)	4(26.67%)	0.361
Anesthesia duration(min)	273.93±57.90	281.40±80.57	0.772
Operation duration (min)	210.13±50.39	223.27±76.34	0.596
ICU stay time (h)	41(21-90)	44(19-93)	0.744
AKI, n (%)	5(33.33%)	6(40%)	0.705

Data are expressed as numbers (percentages) for categorical variables and mean±SD or median (25th-75th percentile) for continuous variables, as appropriate. Abbreviations: BMI, body mass index; CHD, coronary heart disease; PVD, peripheral vascular disease; LVEF, left ventricular ejection fraction; RWMA, regional wall motion abnormality; CPB, cardiopulmonary bypass; AKI, acute kidney injury.

**Table 2:** Comparison of clinical characteristics of patients in BNP-high group and BNP-stable group.

Variables	BNP-high	BNP-stable		*P-*value
Pre-surgical				
SG	1.018±0.012	1.025±0.012	0.113	
K (mmol·L^-1^)	3.98±0.32	3.84±0.34	0.278	
Na (mmol·L^-1^)	142.93±2.17	141.78±3.05	0.247	
Creatinine (μmol·L^-^1)	73.86±31.61	77.17±8.10	0.698	
NT-proBNP (pg·ml^-^1)	49.70(34.40-122)	444(348-631)	<0.001^*^	
CK-MB (ng·ml^-1^)	0.64(0.56-1.25)	0.86(0.63-1.64)	0.267	
CK (U·L^-^1)	62.93±27.87	67.60±19.66	0.615	
HsTnI (ng·ml^-1^)	0.006(0.003-0.028)	0.014(0.006-0.028)	0.250	
LDH (U·L^-1^)	160.93±36.40	192.87±41.43	0.033^*^	
IGF1 (ng·ml^-1^)	30.01±1.99	28.18±3.60	0.096	
CRP (mg·L^-1^)	2061.58±1164.12	2353.80±1024.79	0.472	
IL6 (pg·ml^-1^)	14.52±9.85	17.63±11.44	0.432	
IL8 (pg·ml^-1^)	477.91±261.23	675.24±513.19	0.195	
IL10(pg·ml^-1^)	416.72±233.46	529.13±312.75	0.274	
TNFα (pg·ml^-1^)	263.58±165.40	272.92±107.68	0.856	
HsCRP (mg·L^-^1)	2.79±3.62	2.51±2.89	0.816	
After-surgery				
SG	1.022±0.016	1.014±0.011	0.089	
K (mmol·L^-^1)	4.25±0.36	4.27±0.26	0.836	
Na (mmol·L^-^1)	141.39±2.55	140.79±2.03	0.484	
Creatinine (μmol·L^-1^)	70.56±19.84	68.05±7.47	0.650	
Maximum creatinine value within 48 hours (μmol·L^-1^)	95.01±31.22	101.38±18.58	0.502	
Highest creatinine value in 7 days (μmol·L^-1^)	97.03±30.32	101.71±18.51	0.614	
CK-MB (ng·ml^-1^)	54.81±68.94	87.41±148.71	0.448	
CK (U·L^-^1)	326.67±102.44	382.67±188.42	0.321	
HsTnI (ng·ml^-1^)	1.215(0.532-3.598)	1.919(0.987-2.871)	0.595	
LDH (U·L^-1^)	276.33±91.38	318±128	0.314	
IGF1 (ng·ml^-^1)	16.95±4.47	19.65±6.75	0.206	
CRP (mg·L^-1^)	2104.58±1017.08	1885.69±929.88	0.543	
IL6 (pg·ml^-1^)	16.42(12.45-20.93)	15.96(7.25-20.70)	0.567	
IL8 (pg·ml^-1^)	623.54±495.09	490.54±218.36	0.775	
IL10 (pg·ml^-1^)	447.48±226.57	376.92±141.76	0.315	
TNFα (pg·ml^-1^)	310.54(238.25-423.25)	213.25(143.25-335.75)	0.089	
HsCRP (mg·L^-^1)	13.02(11.88-13.62)	12.35(11.97-13.01)	0.161	
				

Data are expressed as numbers (percentages) for categorical variables and mean±SD or median (25th-75th percentile) for continuous variables, as appropriate. ^*^P<0.05 comparison between BNP-high group and BNP-stable group. Abbreviations: SG, urine specific gravity; NT-proBNP, N-terminal pro-B type natriuretic peptide; CK-MB, Creatine kinase isoenzymes; CK, Creatine kinase; hsTnI, High sensitivity troponin I; CRP, C-reactive protein; IL6, Interleukin-6; IL8, Interleukin-8; IL10, Interleukin-10; TNFα, Tumor necrosis factor-α; hsCRP, High sensitivity C-reactive protein

**Table 3:** Spearman's rank correlation coefficient (rho) for BNP multiple and other variables

Variables	*r/P/N*	BNP multiple	popSG/preSG	CRP T5/T1	IL6 T5/T1	IL8 T5/T1	IL10 T5/T1	TNFα T5/T1
BNP multiple	*r*	1	0.25	0.172	0.237	0.2	0.355	0.203
	*P*	.	0.182	0.364	0.207	0.288	0.054	0.282
popSG/preSG	*r*	0.25	1	0.187	0.087	0.104	0.165	0.139
	*P*	0.182	.	0.323	0.646	0.585	0.384	0.462
	N	30	30	30	30	30	30	30
CRPT5/T1	*r*	0.172	0.187	1	0.549**	0.121	0.544**	0.610**
	*P*	0.364	0.323	.	0.002	0.523	0.002	<0.001
IL6 T5/T1	*r*	0.237	0.087	0.549**	1	0.167	.597**	0.683**
	*P*	0.207	0.646	0.002	.	0.379	<0.001	<0.001
	N	30	30	30	30	30	30	30
IL8 T5/T1	*r*	0.2	0.104	0.121	0.167	1	0.321	.407*
	*P*	0.288	0.585	0.523	0.379	.	0.083	0.026
IL10 T5/T1	*r*	0.355	0.165	0.544**	0.597**	0.321	1	.574**
	*P*	0.054	0.384	0.002	<0.001	0.083	.	0.001
	N	30	30	30	30	30	30	30
TNFαT5/T1	*r*	0.203	0.139	0.610**	0.683**	0.407*	0.574**	1
	*P*	0.282	0.462	<0.001	<0.001	0.026	0.001	.
	N	30	30	30	30	30	30	30

**Correlation is significant at the 0.01 level; *correlation is significant at the 0.05 level. T1, pre-surgical; T5, after-surgery 24 hours; Abbreviations: pop, after-surgery; pre, pre-surgery; SG, Urine specific gravity; CRP, C-reactive protein; IL6, Interleukin-6; IL8, Interleukin-8; IL10, Interleukin-10; TNFα, tumor necrosis factor-α.

**Table 4:** The common differential lncRNA and miRNA in BNP grouping and AKI grouping

miRNA		lncRNA	
miRNA name	UP/DOWN regulation	lncRNA name	UP/DOWN regulation
hsa-miR-135a-5p	DOWN	MSTRG.129293.3^*^	DOWN
hsa-miR-138-5p	DOWN	MSTRG.129696.10^*^	DOWN
hsa-miR-143-3p	UP	MSTRG.129696.18^*^	DOWN
hsa-miR-206	UP	MSTRG.39610.1^*^	DOWN
hsa-miR-208b-3p	UP	NONHSAT160556.1	DOWN
hsa-miR-223-5p	UP	NONHSAT182156.1	DOWN
hsa-miR-499a-5p	UP	NONHSAT242152.1	DOWN

^*^ The novel lncRNA.
