# Supplementary material for: Identifying Water-Salt Homeostasis and Inflammatory Response in Pathological Cardiac Surgery-Associated Acute Kidney Injury: NT-proBNP-related lncRNAs and miRNAs as Novel Diagnostic Biomarkers and Therapeutic Targets
Source: Int J Med Sci. 2025 Jan 21;22(4):845–55. doi: 10.7150/ijms.107589 (PMC11843142; doi:10.7150/ijms.107589)
Supplement: Supplementary file 1 — Supplementary figures and tables. [file ijmsv22p0845s1.pdf]

Supplemental Material

Table S1. Comparison of postoperative and preoperative ratios of the biochemical indexes in BNP high group and BNP stable group.

Variables	BNP-high	BNP-stable	*P*-value
Water and salt metabolism			
popSG/preSG	1.004±0.02	0.989±0.016	0.030 *
popK/preK	1.074±0.122	1.122±0.140	0.330
popNa/preNa	0.989±0.022	0.993±0.022	0.683
pop48hNa/ pop24hNa	1.005±0.010	1.007±0.010	0.38
pop48hNa/preNa	1.02±0.24	1.03±0.03	0.653
Renal function			
popCr/preCr	2.80±7.27	0.89±0.11	0.325
pop48Cr/preCr	3.61±9.16	1.32±0.24	0.389
pop7dayCr/preCr	3.64±9.15	1.32±0.24	0.624
popeGFR/ pre-eGFR	1.06±0.37	1.14±0.13	0.624
pop7dayeGFR/pre-eGFR	0.76±0.07	0.78±0.03	0.345
Cardiac function			
BNP multiple	9.69(8.11-12.81)	1.19(0.91-1.47)	< 0.001*
popCKMB/preCKMB	64.40±94.12	77.41±130.51	0.775
popCK/preCK	6.21±3.03	6.08±3.10	0.744
pophsTnI / prehsTnI	17.91±21.56	13.09±12.77	0.463
popLDH/ preLDH	1.76±0.61	1.65±0.60	0.512
pop24hIGF1/preIGF1	0.53(0.49-0.56)	0.57(0.50-0.96)	0.325
Inflammatory factor			
pop24hCRP//preCRP	2.07±3.13	1.00±1.06	0.148
pop24hIL6/preIL6	2.69±4.65	1.11±0.54	0.174
pop24hIL8/preIL8	1.46±0.97	1.97±3.89	0.217
pop24hIL10/preIL10	1.10(0.79-1.50)	0.69(0.55-0.87)	0.021*
pop24hTNFα/preTNFα	1.97±1.77	0.97±0.50	0.023*
pop hsCRP /pre hsCRP	17.91±21.56	13.09±12.77	0.624

Data are expressed as number (percentage) for categorical variables and mean±SD or median (25th-75th percentile) for continuous variables, as appropriate. pre, preoperative; pop, postoperative; pop24h, postoperative 24 hours; pop48h, postoperative 48 hours

*$P < 0.05$ comparison between BNP-high group and BNP-stable group

Abbreviations: SG, urine specific gravity; eGFR, estimated glomerular filtration rate; BNP, B type natriuretic peptide; BNP multiple=popNT-proBNP/preNT-proBNP; CK-MB, creatine kinase isoenzymes; CK, creatine kinase; hsTnI, high sensitivity troponin I; LDH, lactic dehydrogenase; IGF1, Insulin-like growth factor 1; CRP, C-reactive protein; IL6, Interleukin-6; IL8, Interleukin-8; IL10, Interleukin-10; TNFα, tumor necrosis factor-α, hsCRP, high sensitivity C-reactive protein.

Table S2. QC results of miRNA sequencing and lncRNA sequencing

QC results of miRNA sequencing

Sample Name	Seq. Type	Clean Reads	Q20 Value	Result
1124807-pop24h	miRNA seq	33768382	98.90%	Pass
1124807-Pre-Surg	miRNA seq	22675395	99.23%	Pass
1183893-pop24h	miRNA seq	19174136	99.12%	Pass
1183893-Pre-Surg	miRNA seq	36574937	98.89%	Pass
1266237-pop24h	miRNA seq	37184450	98.41%	Pass
1266237-Pre-Surg	miRNA seq	32950833	99.28%	Pass
1269904-pop24h	miRNA seq	39281194	98.34%	Pass
1269904-Pre-Surg	miRNA seq	31027516	99.24%	Pass
1275157-pop24h	miRNA seq	36818567	98.40%	Pass
1275157-Pre-Surg	miRNA seq	38383301	98.91%	Pass
1275218-pop24h	miRNA seq	32477027	98.32%	Pass
1275218-Pre-Surg	miRNA seq	24950614	99.15%	Pass
1275455-pop24h	miRNA seq	14137830	98.49%	Pass
1275455-Pre-Surg	miRNA seq	21662733	99.04%	Pass
1277426-pop24h	miRNA seq	36282984	98.97%	Pass
1277426-Pre-Surg	miRNA seq	27948269	99.21%	Pass
1277673-pop24h	miRNA seq	32534042	98.81%	Pass
1277673-Pre-Surg	miRNA seq	27718083	0.9907	Pass
1280960-pop24h	miRNA seq	28783764	98.93%	Pass
1280960-Pre-Surg	miRNA seq	27540407	99.20%	Pass
1293840-pop24h	miRNA seq	19804677	98.58%	Pass
1293840-Pre-Surg	miRNA seq	52298633	98.93%	Pass
1296647-pop24h	miRNA seq	32640020	98.99%	Pass
1296647-Pre-Surg	miRNA seq	20637168	99.26%	Pass
1297201-pop24h	miRNA seq	30006629	99.19%	Pass
1297201-Pre-Surg	miRNA seq	34543465	98.91%	Pass
1298054-pop24h	miRNA seq	37260582	98.86%	Pass
1298054-Pre-Surg	miRNA seq	38128582	98.43%	Pass
1298203-pop24h	miRNA seq	37374200	98.40%	Pass
1298203-Pre-Surg	miRNA seq	20682066	99.00%	Pass
1298311-pop24h	miRNA seq	38557109	98.46%	Pass
1298311-Pre-Surg	miRNA seq	40146024	98.96%	Pass

1299509-pop24h	miRNA seq	35094050	98.85%	Pass
1299509-Pre-Surg	miRNA seq	28111863	99.22%	Pass
1299512-pop24h	miRNA seq	35986034	98.84%	Pass
1299512-Pre-Surg	miRNA seq	32836218	98.84%	Pass
1300478-pop24h	miRNA seq	27009861	99.18%	Pass
1300478-Pre-Surg	miRNA seq	40823825	98.89%	Pass
1301448-pop24h	miRNA seq	28226451	98.81%	Pass
1301448-Pre-Surg	miRNA seq	28767531	99.20%	Pass
1302126-pop24h	miRNA seq	34204521	98.90%	Pass
1302126-Pre-Surg	miRNA seq	35949591	98.88%	Pass
1302827-pop24h	miRNA seq	36615544	99.24%	Pass
1302827-Pre-Surg	miRNA seq	25185559	99.05%	Pass
1303762-pop24h	miRNA seq	28844213	99.24%	Pass
1303762-Pre-Surg	miRNA seq	50260002	98.93%	Pass
1305655-pop24h	miRNA seq	40547915	98.89%	Pass
1305655-Pre-Surg	miRNA seq	33365566	98.84%	Pass
1306634-pop24h	miRNA seq	46146250	99.12%	Pass
1306634-Pre-Surg	miRNA seq	35432238	98.92%	Pass
1307172-pop24h	miRNA seq	39999355	98.87%	Pass
1307172-Pre-Surg	miRNA seq	28760458	0.9909	Pass
1310736-pop24h	miRNA seq	39109782	98.90%	Pass
1310736-Pre-Surg	miRNA seq	26312300	99.21%	Pass
1314656-pop24h	miRNA seq	29260491	98.72%	Pass
1314656-Pre-Surg	miRNA seq	41913770	98.87%	Pass
718050-pop24h	miRNA seq	42816411	98.64%	Pass
718050-Pre-Surg	miRNA seq	30103018	0.9898	Pass

QC results of lncRNA sequencing

Sample name	Seq_type	Raw bases(G)	Q20 ratio (%)	Result
1124807-pop24h	mRNA	9.87	0.9632	Pass
1124807-Pre-Surg	mRNA	9.51	0.9352	Pass
1183893-pop24h	mRNA	9.15	0.9587	Pass
1183893-Pre-Surg	mRNA	9.68	0.9476	Pass
1266237-pop24h	mRNA	9.75	0.9498	Pass
1266237-Pre-Surg	mRNA	9.2	0.9442	Pass
1269904-pop24h	mRNA	9.53	0.9031	Pass
1269904-Pre-Surg	mRNA	5.53	0.9226	Pass
1275157-pop24h	mRNA	10.22	0.9035	Pass
1275157-Pre-Surg	mRNA	9.75	0.945	Pass
1275218-pop24h	mRNA	11.51	0.8815	Pass
1275218-Pre-Surg	mRNA	9.68	0.9371	Pass
1275455-pop24h	mRNA	3.34	0.9317	Pass
1275455-Pre-Surg	mRNA	9.39	0.9402	Pass
1277426-pop24h	mRNA	10.42	0.9505	Pass
1277426-Pre-Surg	mRNA	9.69	0.9331	Pass
1277673-pop24h	mRNA	9.64	0.9222	Pass
1277673-Pre-Surg	mRNA	9.99	0.9055	Pass
1280960-pop24h	mRNA	9.78	0.9439	Pass
1280960-Pre-Surg	mRNA	10.14	0.9488	Pass
1293840-pop24h	mRNA	7.17	0.93	Pass
1293840-Pre-Surg	mRNA	9.63	0.9566	Pass
1296647-pop24h	mRNA	12.97	0.9664	Pass
1296647-Pre-Surg	mRNA	21.95	0.9563	Pass
1297201-pop24h	mRNA	9.82	0.9375	Pass
1297201-Pre-Surg	mRNA	10.29	0.957	Pass
1298054-pop24h	mRNA	9.83	0.9542	Pass
1298054-Pre-Surg	mRNA	8.78	0.8951	Pass
1298203-pop24h	mRNA	10.75	0.8944	Pass
1298203-Pre-Surg	mRNA	9.98	0.9336	Pass
1298311-pop24h	mRNA	10.03	0.8859	Pass
1298311-Pre-Surg	mRNA	10.02	0.9437	Pass
1299509-pop24h	mRNA	9.78	0.9511	Pass
1299509-Pre-Surg	mRNA	9.29	0.937	Pass
1299512-pop24h	mRNA	5.31	0.9433	Pass
1299512-Pre-Surg	mRNA	9.52	0.956	Pass
1300478-pop24h	mRNA	9.46	0.9599	Pass
1300478-Pre-Surg	mRNA	9.89	0.9574	Pass

1301448-pop24h	mRNA	9.02	0.9446	Pass
1301448-Pre-Surg	mRNA	9.62	0.9342	Pass
1302126-pop24h	mRNA	9.71	0.9429	Pass
1302126-Pre-Surg	mRNA	9.56	0.9461	Pass
1302827-pop24h	mRNA	9.76	0.9378	Pass
1302827-Pre-Surg	mRNA	9.24	0.9014	Pass
1303762-pop24h	mRNA	9.84	0.9449	Pass
1303762-Pre-Surg	mRNA	9.77	0.9584	Pass
1305655-pop24h	mRNA	9.78	0.9431	Pass
1305655-Pre-Surg	mRNA	9.91	0.9529	Pass
1306634-pop24h	mRNA	9.9	0.9441	Pass
1306634-Pre-Surg	mRNA	10.1	0.9545	Pass
1307172-pop24h	mRNA	5.65	0.9481	Pass
1307172-Pre-Surg	mRNA	13.73	0.9069	Pass
1310736-pop24h	mRNA	9.18	0.9496	Pass
1310736-Pre-Surg	mRNA	11.05	0.9485	Pass
1314656-pop24h	mRNA	7.38	0.9222	Pass
1314656-Pre-Surg	mRNA	9.11	0.947	Pass
718050-pop24h	mRNA	9.15	0.9011	Pass
718050-Pre-Surg	mRNA	5.59	0.9416	Pass

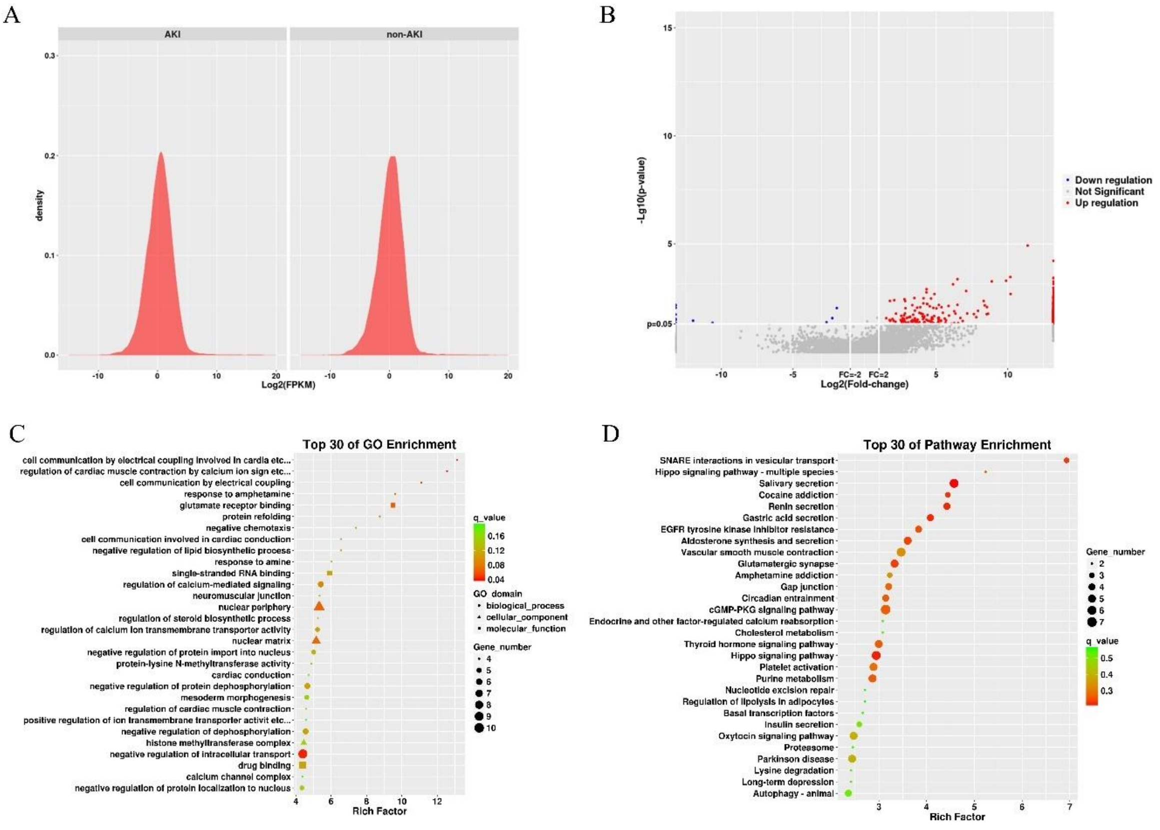

Figure S1. The differential lncRNA of AKI grouping. (B) volcano plot of differential lncRNA. (C) GO term enrichment plot of differential lncRNA target genes. (D) KEGG pathway enrichment plot of differential lncRNA target genes.

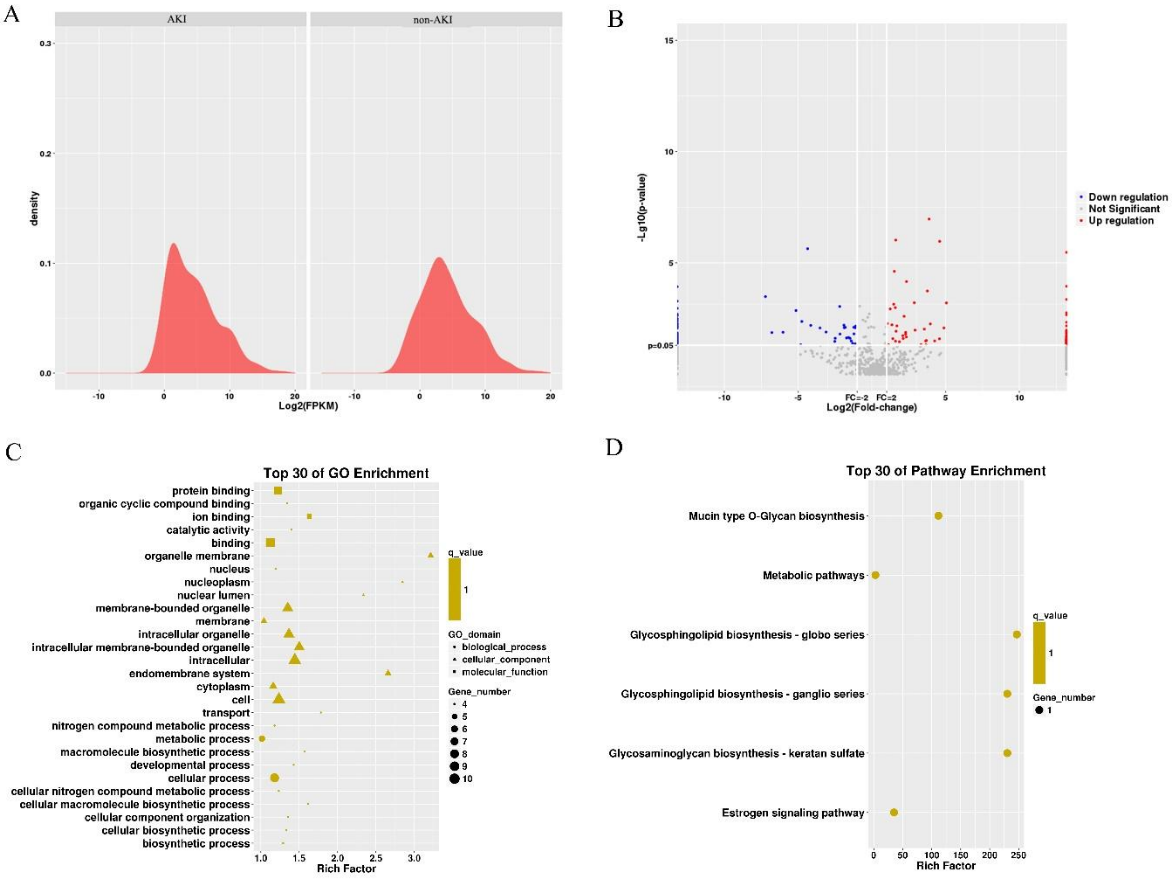

Figure S2. The differential miRNA of AKI grouping. (B) volcano plot of differential miRNA. (C) GO term enrichment plot of differential miRNA target genes. (D) KEGG pathway enrichment plot of differential miRNA target genes.
